# Behind the curtain: unveiling the challenges faced by daily wage teachers in Khyber Pakhtunkhwa universities, Pakistan

**DOI:** 10.3389/fsoc.2024.1471565

**Published:** 2025-03-13

**Authors:** Umar Daraz, Younas Khan, Rula Odeh Alsawalqa, Maissa N. Alrawashdeh, Ann Mousa Alnajdawi, Tariq Aziz

**Affiliations:** ^1^Department of Sociology, University of Malakand, Chakdara, Khyber Pakhtunkhwa, Pakistan; ^2^Department of Sociology and Criminology, Kohat University of Science and Technology, Kohat, Pakistan; ^3^Department of Sociology, The University of Jordan, Amman, Jordan; ^4^Department of Social Work, The University of Jordan, Amman, Jordan

**Keywords:** daily wage teachers, economic instability, academic stagnation, administrative inefficiencies, social ostracization, psychological distress

## Abstract

This study examines the multifaceted challenges faced by daily wage teachers in Khyber Pakhtunkhwa, Pakistan, encompassing social, economic, academic, administrative, and psychological dimensions. The primary objective is to analyze how systemic barriers perpetuate precarious employment conditions and to propose actionable solutions for improving their wellbeing and professional experiences. Social exclusion and inadequate support within academic settings foster feelings of isolation, while the economic instability of per-class payment models exacerbates financial insecurity, leading to diminished morale. Academically, career stagnation persists as these teachers' qualifications and contributions are often undervalued. Additionally, administrative inefficiencies and exploitative practices erode trust and exacerbate their precarious status. Psychological challenges, including heightened anxiety and depression, are prevalent among this population. Using a phenomenological approach, the study employs purposive sampling, semi-structured interviews, and focus group discussions to explore these issues. Thematic analysis reveals the systemic barriers reinforcing the marginalization, financial instability, and administrative hurdles experienced by daily wage teachers. Key findings emphasize the need for targeted reforms, such as mentorship programs to enhance social integration, stable salary structures to ensure financial security, clear pathways for career progression, streamlined administrative processes, and accessible mental health support. The study highlights the urgency of addressing these challenges through inclusive and evidence-based policy measures, advocating for structural changes to improve working conditions and foster equity within the academic landscape.

## Introduction

In Khyber Pakhtunkhwa's higher education system, daily wage teachers play a pivotal role in shaping future generations and advancing societal knowledge. However, systemic challenges within this sector impose significant barriers that hinder their professional growth and personal wellbeing (Rasheed et al., 2016). Among these challenges, psychological distress stands out as a critical concern, with elevated levels of anxiety, depression, and insecurity commonly reported. Precarious employment conditions, compounded by social exclusion and financial instability, exacerbate these mental health struggles, undermining both morale and job performance (Rose, 2014; Dutta, 2023). Such psychological impacts extend beyond individual wellbeing, affecting educators' capacity to contribute positively to academic environments.

Social exclusion and marginalization further intensify these psychological challenges. Research from Pakistan indicates that the temporary and contingent nature of employment often relegates daily wage teachers to the periphery, subjecting them to isolation and limited professional engagement. In academic settings, this marginalization diminishes their sense of belonging and professional identity, as they are frequently viewed as transient contributors (Arif and Ilyas, 2013; Pasha, 2022).

Economic instability presents another pervasive issue. The per-class payment model often fails to meet financial needs, leaving daily wage teachers in a precarious position. Studies highlight that financial insecurity is exacerbated by bureaucratic delays in reimbursements and administrative inefficiencies, further eroding morale and commitment (Ali, 2018; Ahtesham, 2024).

Despite possessing the necessary qualifications, many daily wage teachers face academic stagnation. Institutional reluctance to facilitate career advancement or provide necessary documentation, such as no-objection certificates, restricts their professional mobility (Knight, 2002; Ali et al., 2024). Similar challenges are reported globally, where adjunct faculty face job insecurity, limited progression opportunities, and adverse impacts on mental health and professional development (Anjum, 2021; Rhoades et al., 2024).

Administrative inefficiencies compound these issues. Arbitrary decision-making and a lack of transparency in administrative processes expose daily wage teachers to exploitation, undermining trust in institutional structures (Day et al., 2007; Khan, 2022). This combination of psychological, social, economic, academic, and administrative challenges underscores the systemic barriers that daily wage teachers in Khyber Pakhtunkhwa confront.

This study aims to provide a comprehensive understanding of these interconnected challenges, focusing on the precarious employment conditions of daily wage teachers in the region's higher education landscape. By examining their unique experiences, this research identifies actionable solutions to address their pressing needs. Specifically, the study advocates for policy interventions that foster social integration, ensure fair and consistent compensation, create pathways for career development, streamline administrative processes, and establish mental health support systems. These recommendations aim to inform policymakers, educational administrators, and stakeholders, emphasizing the urgency of structural reforms to promote equity, inclusion, and sustainability within academic institutions. This research not only contributes to the broader discourse on contingent academic labor but also highlights the essential role of daily wage teachers in ensuring the vitality of higher education.

## Literature review

Contingent faculty in higher education face multifaceted challenges globally. While these issues are extensively documented in various contexts, the experiences of such faculty in Pakistan, particularly in Khyber Pakhtunkhwa (KP) universities, remain underexplored. This review examines the social, economic, academic, administrative, and psychological dimensions of these challenges, highlighting their unique manifestations and impacts in KP.

## Social challenges

Globally, contingent faculty often experience marginalization and alienation within academic communities. Hall (2018) and De Welde and Stepnick (2023) report that in the UK, and Jiang (2024) in China, temporary lecturers are frequently excluded from academic networks, limiting meaningful interactions with peers and students and straining professional relationships. In Latin America, studies by Hümbelin (2021) emphasize the stigma associated with non-permanent employment, fostering feelings of disconnection and isolation.

In KP universities, similar patterns emerge. Daily wage teachers often report detachment from their academic communities due to their temporary status, which inhibits the formation of lasting professional relationships (Rasheed et al., 2016). Cultural dynamics and institutional structures in Pakistan may further exacerbate these barriers. Despite these observations, there is a lack of focused research on how social exclusion uniquely affects daily wage teachers in KP, indicating a critical gap in the literature.

## Economic challenges

Economic precarity is a significant issue for contingent faculty worldwide. In Brazil, Castro (2022) and Junge (2018) highlight low wages, irregular income, and limited benefits as key challenges, while Ang (2018) emphasizes systemic inequalities in Japan that deepen financial disparities.

In KP, daily wage teachers face similar financial hardships. Payment structures based on per-class remuneration fail to provide economic stability or meet basic living expenses. Delays in reimbursement, a common issue in Pakistani universities, further exacerbate these challenges (Ali, 2018; Ang, 2018). These financial instabilities often force educators into precarious living conditions. While these economic issues align with global trends, the interplay of local bureaucratic inefficiencies in KP with these challenges warrants deeper investigation.

## Academic challenges

Professional stagnation and career immobility are recurring themes for contingent faculty. Studies in the US by Birdsell Bauer (2011) and in Spain by Burton and Bowman (2022) reveal that temporary lecturers face limited opportunities for career advancement, often excluded from institutional decision-making and professional development initiatives.

In KP, daily wage teachers encounter similar hurdles. Despite their qualifications, their expertise is frequently overlooked, limiting access to permanent positions or professional growth opportunities (Aziz et al., 2014; Naviwala, 2016). Recent research, such as that by Siddiqi (2022) and Murtaza and Hui (2021), indicates that many daily wage teachers in KP are excluded from training programs and research initiatives. Furthermore, Hepler (2021) highlight institutional reluctance to integrate temporary faculty into policymaking, exacerbating feelings of alienation and stagnation.

## Administrative challenges

Administrative inefficiencies often hinder the autonomy and growth of contingent faculty. Globally, Manning (2017) underscores the prevalence of arbitrary treatment, while Reamer (2018) in Australia and Shen (2012) in Taiwan link inefficient administrative systems to exploitation.

In KP universities, similar challenges are evident. Delayed salary disbursements and cumbersome reimbursement processes are common, as noted by Arif and Ilyas (2013). Shahzad et al. (2008) emphasize the discretionary powers of administrators, often resulting in inconsistent and exploitative treatment. Recent studies, including those by Tahira et al. (2020) and Taj et al. (2021), reveal a lack of grievance redressal mechanisms for contingent faculty, leaving them vulnerable to exploitation. The absence of transparent hiring practices further erodes trust in institutional systems, perpetuating administrative challenges.

## Psychological wellbeing

The psychological wellbeing of contingent faculty is deeply affected by the challenges they face. In the US, Gillespie et al. (2001) and Blustein et al. (2019) document widespread anxiety, stress, and disillusionment among temporary lecturers. Similarly, Szkudlarek et al. (2021) in South Korea and Stevens (2019) in Vietnam highlight the mental health repercussions of precarious employment.

In KP universities, the psychological distress of daily wage teachers stems from intersecting challenges. Social exclusion within academic communities fosters isolation and reduces morale (Rasheed et al., 2016). Economic precarity, characterized by low wages and delayed payments, amplifies stress and uncertainty (Naviwala, 2016; Ali et al., 2022). Administrative inefficiencies and exploitation further contribute to feelings of vulnerability (Azhar et al., 2024; Qadir and Afzal, 2024). These overlapping stressors—such as delayed salaries (economic challenge), lack of institutional support (administrative challenge), and social isolation—compound psychological strain, manifesting in heightened anxiety and depression.

## Gaps in the literature

While this review integrates insights from global and local studies, there remains a pressing need for focused research on the unique challenges faced by daily wage teachers in KP. Specifically, the interplay of social, economic, academic, and administrative factors with psychological wellbeing is insufficiently explored. Addressing these gaps will provide a clearer understanding of the systemic barriers faced by contingent faculty in KP and guide targeted interventions to improve their working conditions and overall wellbeing.

## Study problem

Daily wage teachers in Khyber Pakhtunkhwa (KP), Pakistan, face multifaceted challenges that hinder their professional growth and wellbeing. These challenges span social, economic, academic, administrative, and psychological domains, manifesting in social marginalization, financial instability, limited career advancement opportunities, bureaucratic inefficiencies, and heightened psychological stress. Despite their critical role in the higher education system, these educators' experiences remain largely neglected in academic research.

Global studies have highlighted issues such as exclusion, financial insecurity, career stagnation, and mental health struggles among contingent faculty in higher education (Hall, 2018; De Welde and Stepnick, 2023; Castro, 2022; Burton and Bowman, 2022). However, there is limited research on how the socio-economic and institutional conditions specific to KP exacerbate these issues for daily wage teachers. In KP, low per-class payments often fail to meet basic living expenses, while bureaucratic delays in reimbursements heighten financial stress and uncertainty. Contractual arrangements typically provide minimal job security, health benefits, or social protections. Frequently classified as “self-employed,” these educators are excluded from labor rights extended to permanent employees (Rasheed et al., 2016; Arif and Ilyas, 2013).

This study seeks to provide a comprehensive analysis of the challenges faced by daily wage teachers in KP universities, aiming to inform policy and institutional reforms. Key recommendations include ensuring fair compensation, providing job security, and revising employment classifications to grant educators benefits comparable to permanent staff, such as health insurance, pensions, and paid leave.

Addressing these gaps, this research offers a localized perspective on the struggles of daily wage teachers in KP universities, emphasizing the role of regional socio-economic and institutional dynamics in shaping their experiences. By focusing on the socio-economic, administrative, and psychological dimensions of their challenges, the study aims to guide policymakers, institutional stakeholders, and advocacy groups. Targeted reforms in compensation, job security, and labor rights will contribute to systemic improvements, enhancing the professional conditions and wellbeing of daily wage teachers while reinforcing their pivotal role in higher education.

## Socioeconomic context and timeline

**Figure 1:**
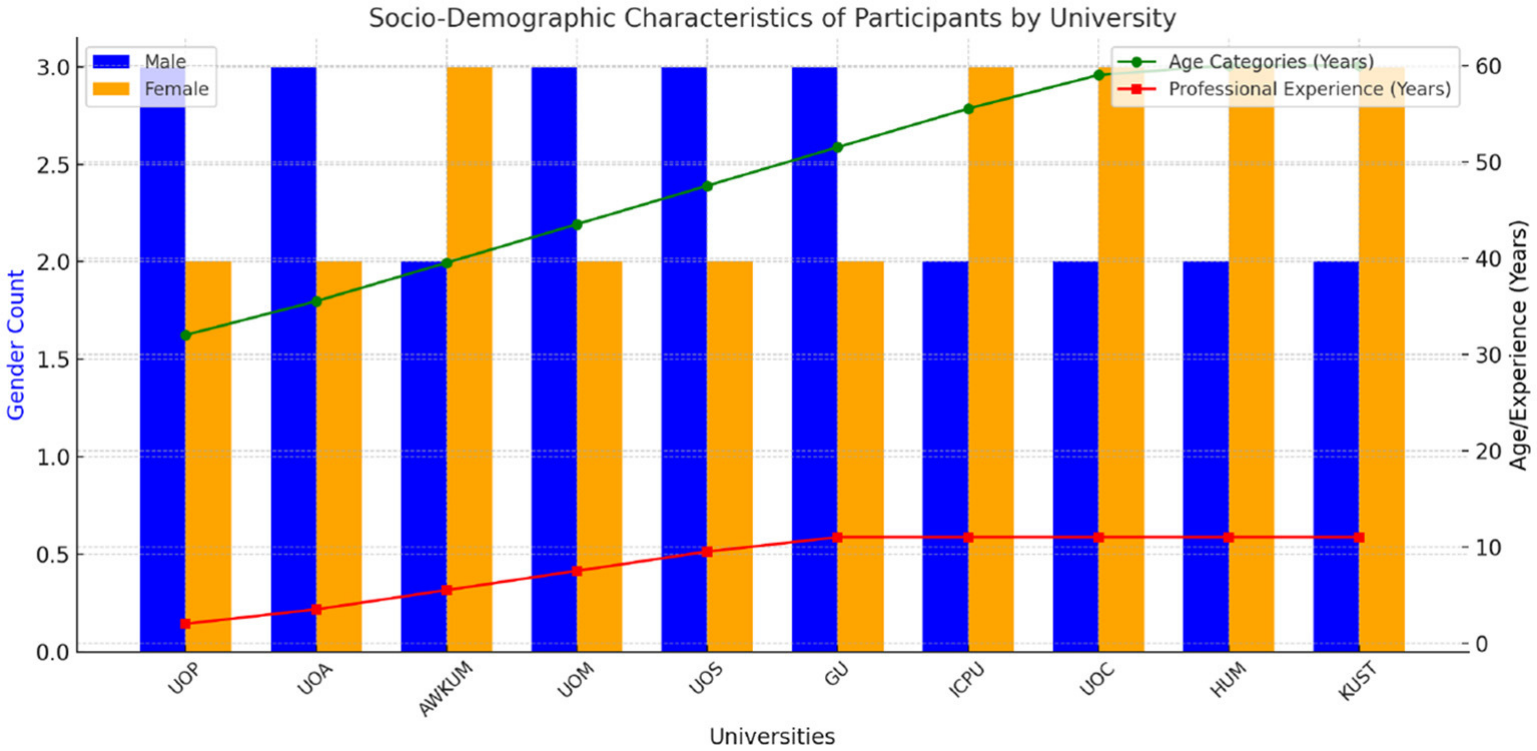
Socio-demographic characteristics of participants by university.

The challenges faced by daily wage teachers in KP universities must be understood within the broader socio-economic context of Pakistan. The country's education sector, particularly in regions like KP, faces systemic issues, including inadequate funding, administrative inefficiencies, and inconsistent policy implementation. These challenges are further compounded by Pakistan's economic instability, marked by high inflation, unemployment, and constrained public spending (see Figure 1).

The government's limited education budget often prioritizes infrastructure and administrative expenses over faculty development and welfare. Consequently, daily wage teachers—employed on temporary contracts and paid per class—remain especially vulnerable. Their precarious employment conditions reflect broader labor market trends in Pakistan, where informal and insecure jobs are prevalent. This workforce segment typically lacks social security benefits, job stability, and opportunities for professional growth, mirroring the widespread issues of underemployment and labor exploitation in the country.

## Timeline of challenges (2010–2024)

The analysis spans 2010 to 2024, a period of significant developments in Pakistan's higher education sector, characterized by increased reliance on daily wage teachers due to budgetary constraints and growing demand for higher education.

### 2010–2014

Following the 18th Amendment, education became a provincial subject, leading to variations in commitment and resource allocation for higher education across provinces. KP witnessed a gradual increase in universities and higher education institutions, driving a higher demand for teaching staff.

### 2015–2019

This period marked a dual trend of higher education sector expansion and financial strain. Universities increasingly relied on daily wage teachers to accommodate rising student enrollments without corresponding increases in funding. While some policy initiatives aimed to improve faculty conditions, their implementation remained inconsistent.

### 2020–2024

The COVID-19 pandemic exacerbated existing challenges, leading to budget cuts and a greater dependence on temporary teaching staff. Financial insecurities worsened for daily wage teachers amid economic instability. The shift to digital and remote learning brought new challenges and opportunities, further complicating the landscape for temporary faculty.

## Methodology

### Study design

This study employed a phenomenological research design to explore the lived experiences of daily wage teachers in Khyber Pakhtunkhwa (KP) universities. This approach is particularly appropriate for understanding the essence of individual experiences within a specific phenomenon, enabling a nuanced examination of the socio-economic, academic, administrative, and psychological challenges faced by these educators (Creswell and Poth, 2016). Phenomenology focuses on capturing subjective perceptions and emotional dimensions, such as stress, anxiety, and marginalization, which are central to understanding precarious employment in academic settings (Smith et al., 2021).

Phenomenology was chosen over other qualitative methods, such as grounded theory and ethnography, because of its emphasis on understanding individual lived experiences rather than generating theoretical frameworks or analyzing cultural patterns. While grounded theory develops process-oriented theories and ethnography immerses itself in cultural contexts, these approaches are less aligned with the study's aim of interpreting subjective realities. Phenomenology provides the depth needed to uncover how daily wage teachers perceive and navigate their challenges, offering insights that broader quantitative studies might overlook.

Rooted in Husserl's tradition, the study adopts a descriptive phenomenological approach, emphasizing bracketing to minimize researcher bias and authentically portray participants' perspectives (Vagle, 2018). This approach allows for a clear depiction of themes such as financial instability, job insecurity, and psychological distress, ensuring participants' voices are authentically represented. By avoiding interpretive overlays, the study provides an unbiased account of the systemic factors influencing these educators' professional and personal lives.

The methodological choice aligns with the study's objectives by facilitating a detailed exploration of the multifaceted challenges daily wage teachers face, including economic precarity, social marginalization, and institutional barriers. Bracketing ensures a faithful representation of these experiences, while in-depth interviews reveal the interplay between socio-economic pressures and administrative practices. This design supports the goal of identifying root causes of these challenges, providing an empirical foundation for policy reforms aimed at addressing systemic inequities in higher education institutions (Van Manen, 2016).

Phenomenology has been widely recognized as an effective approach in education and employment contexts, particularly for exploring individual navigation of complex institutional realities. Its focus on lived experiences makes it well-suited to uncovering insights into the challenges faced by daily wage teachers and informing meaningful reforms (Creswell and Poth, 2016; Van Manen, 2016).

### Universe and participants

This study investigates the challenges faced by daily wage teachers in Khyber Pakhtunkhwa universities, targeting institutions such as the University of Peshawar (UOP), Abdul Wali Khan University Mardan (AWKUM), University of Malakand (UOM), University of Swat (UOS), and Hazara University Mansehra (HUM), among others. Selection criteria included institutional size, funding structure, geographic distribution, and a significant presence of daily wage teachers. By incorporating both well-established and newer universities, the study ensures a balanced understanding of the challenges across diverse institutional settings. For example, larger universities like UOP provide insights into resource-intensive environments, while smaller or developing institutions such as the University of Chitral (UOC) highlight issues specific to under-resourced contexts. The geographic distribution also allows the study to capture regional variations in socio-economic and administrative dynamics, offering a nuanced perspective on the lived experiences of these educators.

Participants were selected based on specific inclusion and exclusion criteria. Eligible participants were required to be employed as daily wage or visiting teachers in the selected universities, with a minimum tenure of 6 months to ensure adequate experience with systemic challenges. Teachers from both natural sciences and social sciences were included to reflect disciplinary variations in teaching loads, resource allocation, and student-teacher dynamics. Participation was voluntary, emphasizing ethical compliance and ensuring the authenticity of responses. Teachers with < 6 months of tenure, those in permanent or tenure-track positions, and individuals in non-teaching roles were excluded to maintain the focus on the unique challenges faced by daily wage teachers.

A purposive sampling strategy was employed to identify participants through departmental records and recommendations from university networks. This was complemented by snowball sampling, where initial participants referred colleagues in similar roles, expanding the study's reach and incorporating individuals who might otherwise be inaccessible. Departments with significant reliance on daily wage faculty and those representing a range of academic disciplines were prioritized to ensure comprehensive coverage of institutional contexts.

While accessibility challenges limited the inclusion of some participants, particularly those hesitant due to job insecurity, these obstacles underscore areas for future research, such as exploring innovative methods to reach underrepresented voices. Measures to enhance accessibility included leveraging university networks and conducting virtual interviews, while confidentiality assurances and informed consent helped mitigate participants' concerns. Efforts were also made to balance representation across disciplines and institutions, ensuring the findings reflect the diverse realities of daily wage teachers in Khyber Pakhtunkhwa universities. These challenges, rather than detracting from the study, highlight opportunities for further exploration of systemic barriers and their broader implications.

## Socio-demographic characteristics of participants

Participants in this study encompass a diverse demographic profile, including individuals of varying ages, genders, educational backgrounds, and professional experiences. Demographic information such as age, gender, educational qualifications, years of teaching experience, and institutional affiliation is collected to contextualize the findings and ensure diversity within the sample (see Table 1).

**Table 1:** Socio-demographic characteristics of participants.

**University**	**Gender**	**No**.	**Age**	**Educational status**	**Professional experience**
UOP	M/F	3/2	30–33	MS	0–2 Years
UOA	M/F	3/2	34–37	MS	3–4 Years
AWKUM	M/F	3/2	38–41	PhD	5–6 Years
UOM	M/F	2/3	42–45	PhD	7–8 Years
UOS	M/F	2/3	46–49	MS	9–10 Years
GU	M/F	3/2	50–53	MS	10 Plus Years
ICPU	M/F	2/3	54–57	MS	10 Plus Years
UOC	M/F	3/2	58–60	MS	10 Plus Years
HUM	M/F	2/3	-	MS	10 Plus Years
KUST	M/F	3/2	-	MS	10 Plus Years

M, Male; F, Female; Number of respondent = 25.

### Sampling and participant selection

This study utilized purposive sampling, a widely recognized method for qualitative research that seeks to identify participants with direct and relevant experience in the phenomenon under investigation (Patton, 2014; Campbell et al., 2020). This approach ensured the inclusion of individuals uniquely positioned to provide insights into the challenges faced by daily wage teachers in Khyber Pakhtunkhwa universities. The selection criteria required participants to be current daily wage teachers with at least 1 year of professional experience, ensuring familiarity with institutional and administrative dynamics. Gender representation was emphasized, with efforts to achieve a balanced representation of males and females to explore gender-specific challenges. Additionally, participants were drawn from diverse educational (MS/PhD) and age backgrounds to capture varied perspectives across disciplines and institutional contexts.

The researchers initially targeted 50 participants, selecting five from each of the 10 universities. This number aligns with recommendations in the qualitative research literature, such as Fusch Ph and Ness (2015) guidance suggesting 20–50 participants for studies exploring complex phenomena. From a statistical perspective, the target size aimed to balance representational diversity across institutions while being feasible in terms of time and resource constraints. The allocation of five participants per university ensured equal institutional representation while remaining manageable for in-depth qualitative analysis.

Gender was carefully considered during participant selection, with an initial goal of 25 males and 25 females to identify differences in their experiences. Following a reduction to 25 participants due to thematic saturation, gender balance was maintained, with 12 males and 13 females interviewed. Thematic saturation, a critical concept in qualitative research (Guest et al., 2006, 2020), was systematically assessed. After every five interviews, researchers reviewed transcripts to identify recurring themes. By the 25th interview, no new themes emerged, and prominent issues such as job insecurity, financial instability, and limited professional growth were consistently identified (Saunders et al., 2018; Hennink and Kaiser, 2022).

Focus group discussions complemented individual interviews, with one session held per university involving 3–5 participants. This range is supported by the literature (Morgan, 1996), as smaller groups encourage deeper engagement and allow for diverse perspectives within manageable dynamics (Morgan et al., 1998; Hennink and Kaiser, 2022). Gender representation in focus groups was balanced wherever possible, ensuring inclusivity in collaborative discussions. The total number of participants in focus groups was 35, with each session enriched by exploring shared experiences and group dynamics.

Acknowledging potential biases in purposive sampling, such as overrepresentation of accessible or willing participants, the researchers employed diverse recruitment strategies to mitigate this limitation. Methods included outreach through academic networks, union representatives, and advocacy groups, enabling access to a broader pool of candidates, including those less likely to self-select into the study. To address underrepresentation, participants from less visible departments and disciplines were actively sought, informed by consultation with university administration and department heads. Efforts to include these voices ensured a more comprehensive representation of the challenges faced by daily wage teachers across institutional contexts.

These methodological choices, while effective for this study, underscore opportunities for future research. Combining purposive sampling with random sampling could enhance generalizability, providing further validation of the findings and extending their applicability across similar educational contexts.

### Data collection

The study employed semi-structured interviews and focus group discussions with daily wage teachers from selected universities during September 2023. This process aimed to gather comprehensive insights into the multifaceted challenges faced by these educators.

#### Preparation and design

The data collection instruments were designed to capture socio-demographic details and explore challenges across social, economic, academic, administrative, and psychological dimensions. The interview guide underwent rigorous pretesting to ensure clarity and relevance. Adjustments included simplifying questions, encouraging open-ended responses, and removing redundancies.

### Data collection methods

#### Semi-structured interviews

A total of 25 daily wage teachers participated, with sessions lasting 45–60 min. Interviews were conducted in private university spaces or virtually, ensuring a confidential and comfortable setting.

#### Focus group discussions

Ten focus groups, one per university, were conducted with 3–5 participants per group. Discussions lasted 60–90 min, focusing on themes such as economic instability, professional development challenges, and institutional reforms.

### Recording and documentation

All sessions were audio recorded with participant consent and transcribed verbatim. Field notes captured non-verbal cues and contextual observations to enrich the data. Translations were provided in the multilingual context where necessary.

### Credibility and trustworthiness

The study ensured credibility through member checking, peer debriefing, and triangulation. Key points from interviews were verified by participants, and insights from different data collection methods were cross-referenced to enhance reliability and depth.

### Challenges faced

Several challenges arose, including participant accessibility due to mistrust or fear of retaliation, time constraints that limited response depth, and potential nuances lost in translation due to the multilingual context.

### Ethical considerations

The study adhered to ethical standards approved by the Institutional Review Board (Approval No. UOM-IRB-2023-09/02). Participants provided informed consent and were assured of confidentiality and voluntary participation.

### Analytical approach

Thematic analysis was employed to identify recurring ideas, categorize data, and develop overarching themes. This iterative process included coding and validation through participant feedback and peer input to ensure the authenticity of findings.

### Data analysis

In the data analysis phase, a thematic approach was employed to analyze the qualitative data gathered from interviews, focus group discussions, and document analysis. This analysis followed the guidelines provided by Braun and Clarke (2006) and Bryman (2016), ensuring a rigorous, systematic process. The first step of Braun and Clarke's thematic analysis framework, familiarization with the data, involved transcribing all interviews and focus group discussions and repeatedly reading through the data to gain a deep understanding of its content. This allowed the researchers to become intimately familiar with the material, noting initial ideas and patterns as they emerged from the data.

The second step, generating initial codes, involved systematically coding the data. Codes were created based on meaningful segments of text that related to the research objectives. These codes were designed to capture specific elements within the data that related to the challenges faced by daily wage teachers in Khyber Pakhtunkhwa universities. Coding was done manually and using NVivo software to support a structured approach. The codes were developed in an open and inductive manner, allowing for the identification of emergent themes while also remaining aligned with the research questions.

The third step, reviewing themes, was conducted by grouping similar codes into broader themes. These themes were iteratively refined using the constant comparative method, a process that allowed for the continuous comparison of new data with previously collected data. This approach facilitated theme development and refinement by ensuring that new data contributed to an ongoing, dynamic understanding of the challenges faced by daily wage teachers. The constant comparative method helped validate the coding scheme, ensuring consistency and reliability across the coding process.

The thematic analysis involved a systematic coding and categorization process, which was carried out by two researchers independently to ensure reliability. Any disagreements that arose during the coding process were resolved through discussion and consensus. To further enhance the credibility of the findings, a third researcher was consulted to review the final coding structure and validate the categorization process. NVivo software was used to organize and manage the data, enabling the researchers to track codes and themes effectively. This software facilitated a structured analysis and provided a clear audit trail for transparency.

Themes were interpreted in relation to the research objectives, ensuring that the analysis remained focused on the study's aims. Some themes were pre-defined based on the research questions, while others emerged naturally from the data. The research objectives guided the identification and interpretation of the themes, helping to align the emergent findings with the specific challenges that daily wage teachers face in Khyber Pakhtunkhwa universities. The final interpretation of the themes provided rich, descriptive insights into these challenges, supporting the study's goal of understanding the multifaceted nature of the teachers' experiences.

## Limitations of the methodology

### Limited generalizability

The use of purposive and snowball sampling limits the generalizability of findings beyond the specific sample. While this method ensures the inclusion of participants with relevant experiences, it risks over-representing more accessible or willing individuals, potentially missing voices of those less engaged in institutional networks. The focus on universities within Khyber Pakhtunkhwa may not fully capture the diversity of challenges faced by daily wage teachers in other regions or countries.

### Sample size constraints

While the study achieved thematic saturation with 25 participants, this relatively small sample size may overlook less common but significant experiences or perspectives. The focus on thematic saturation risks excluding potentially relevant outlier cases that might enrich the findings.

### Reliance on self-reported data

The study heavily depends on self-reported data from interviews and focus groups, which are subject to biases such as social desirability or selective recall. Participants may downplay or exaggerate their experiences due to fear of retaliation or a desire to influence policy recommendations.

### Translation and multilingual context

Conducting interviews in multiple languages introduces the risk of losing nuances during translation. Important cultural or emotional subtleties may have been diluted, impacting the authenticity of the data.

### Bracketing challenges

While the study employed **bracketing** to minimize researcher bias, achieving complete neutrality is difficult. Researchers' preconceptions and the subjective interpretation of themes may have influenced the analysis, particularly in identifying and grouping emergent themes.

### Access barriers

Some participants were hesitant to engage due to mistrust or job insecurity, potentially leading to an underrepresentation of voices from particularly vulnerable individuals or those in precarious employment. Efforts to mitigate this limitation, such as confidentiality assurances, may not have fully addressed these barriers.

### Focus on institutional contexts

The study focuses primarily on institutional dynamics (e.g., job security, financial instability) and may have underexplored external factors such as broader socio-economic or political influences that shape the lived experiences of daily wage teachers.

### Challenges with focus group dynamics

While focus groups provided collaborative insights, power dynamics among participants (e.g., senior vs. junior teachers) may have influenced the openness of discussions. Dominant voices could have overshadowed less assertive participants, skewing the data.

### Resource limitations

Conducting virtual interviews for accessibility had potential downsides, such as technical issues, reduced engagement, or lack of non-verbal cues, which could have affected the richness of the data.

### Overemphasis on thematic analysis

The thematic analysis framework focused on recurring themes, potentially overlooking isolated but significant experiences that do not fit into broader categories. This approach, while systematic, may miss the depth and complexity of certain individual narratives.

### Ethical dilemmas

Despite assurances of confidentiality, participants' concerns about the potential misuse of data in an academic or institutional context may have influenced the level of openness in their responses.

## Results

### Social challenges

According to respondents daily wage teachers encounter social hurdles like ostracization and strained relationships within academia due to their temporary status. This detachment diminishes their sense of belonging and integration within the academic community.

In this regard, Daily wage teacher-1 argues:

“*I often feel like an outsider, barely acknowledged by my peers. It's disheartening to see students and permanent staff treat us as disposable, hindering any sense of belonging*.”…. [UOPMMS-1].

Similarly, Daily wage teacher-2 explores:

“*I struggle to establish meaningful connections in the academic community. Our temporary status creates a barrier, making it challenging to engage with colleagues and students on a deeper level*.”…. [UOPMPHD-2].

In the same way, Daily wage teacher-3 notes:

“*I constantly grapple with a sense of detachment. It's demoralizing to be seen as a transient figure, devoid of significance beyond fulfilling teaching duties. This lack of integration affects my morale and motivation*.”…. [UOPFMS-3].

While daily wage teacher-4 expresses:

“*I yearn for inclusion and recognition within the university. It's disheartening to be treated as expendable, without any investment in our professional growth or wellbeing. This marginalization takes a toll on our sense of worth*.”…. [UOAMMS-4].

Moreover, daily wage teacher-5 demonstrates:

“*I feel like a peripheral figure in the academic landscape. Our non-permanent status perpetuates a cycle of exclusion, leaving us isolated and undervalued. It's disheartening to be constantly reminded of our transient position*.”…. [UOAFPHD-5].

Daily wage teachers encounter significant social hurdles, with their temporary status leading to ostracization and strained relationships within the academic community. This detachment erodes their sense of belonging and integration, leaving them feeling undervalued and isolated. In focus group discussions, participants collectively emphasized the sense of marginalization. For example, one participant remarked:

“*We often feel invisible in the academic setup. Even our presence in meetings is either overlooked or treated as a formality*.”

Another echoed this sentiment, adding:

“*The stigma around being a 'temporary teacher' creates a barrier to forming meaningful relationships with permanent staff and students*.”

Gender dynamics emerged in focus group discussions, highlighting that female daily wage teachers experience additional social challenges, such as fewer networking opportunities and greater scrutiny. Breaking down the theme into sub-themes like “Peer Ostracization,” “Student Perceptions,” and “Gendered Exclusion” enriches the analysis.

The focus group discussions (FGDs) provided a rich understanding of the collective experiences of daily wage teachers, building upon the themes identified in individual interviews. While interviews highlighted individual struggles with social integration, FGDs brought to light the shared frustration and systemic nature of these challenges.

Both interviews and FGDs underscored the pervasive sense of exclusion faced by daily wage teachers. Participants in FGDs collectively expressed feelings of being sidelined by permanent staff and students. A participant remarked:

“*We often feel invisible in the academic setup. Even our presence in meetings is either overlooked or treated as a formality*.” (FGD, UOP, September, 2023).

Another echoed this sentiment:

“*The stigma of being a ‘temporary teacher' creates an unspoken barrier. It's like we're not really part of the team, no matter how hard we try*.” (FGD, UOS, September 2023).

While interviews revealed individual accounts of ostracization, FGDs emphasized a collective frustration with the systemic lack of acknowledgment.

FGDs revealed a consensus that students often perceived daily wage teachers as less credible or influential compared to permanent faculty. One participant shared:

“*Students tend to treat us differently, as if our temporary status somehow makes us less competent. This perception is deeply demoralizing*.” (FGD, UOA, September 2023).

Interviews had also highlighted this theme, but FGDs added depth by illustrating how these perceptions contribute to a shared sense of professional inadequacy.

Gender dynamics emerged prominently in FGDs, with female participants articulating additional layers of social exclusion. A female participant explained:

“*As a woman, I find it even harder to network or be taken seriously. The opportunities for us to grow are even more limited, and the scrutiny we face is exhausting*.” (FGD, UOMM, November 2023).

Another added:

“*There are fewer informal avenues for us to connect with colleagues. Our male counterparts seem to navigate these barriers more easily*.” (FGD, GU, December, 2023).

While interviews with female teachers touched on these challenges, FGDs highlighted the collective nature of gendered exclusion, offering a more nuanced view of the issue.

Focus group discussions provided a platform for participants to collectively articulate frustrations that interviews presented as isolated struggles. For instance, while interviews emphasized individual feelings of detachment, FGDs revealed a shared sense of professional stagnation. A participant summarized this sentiment:

“*Despite our qualifications, we are treated as expendable labor. It's not just me; all of us feel trapped in a cycle of stagnation*.” (FGD, UOC, December 2023).

FGDs also revealed nuances, such as the distinct challenges faced by female teachers, which were less pronounced in the semi-structured interviews.

The FGDs enriched the analysis by highlighting collective experiences and systemic barriers faced by daily wage teachers. The recurring themes of peer ostracization, student perceptions, and gendered exclusion align closely with findings from individual interviews but add a collective dimension that underscores the broader impact of temporary academic positions. This convergence and divergence between the two data sources provide a comprehensive understanding of the social challenges encountered by daily wage teachers in academia.

### Economic challenges

Daily wage teachers endure economic instability, receiving compensation per class that often fails to suffice for their basic needs. Bureaucratic obstacles delay reimbursements, worsening financial strain and dampening their morale and commitment to teaching.

Moreover, Daily wage teacher-6 recognizes:

“*I constantly worry about making ends meet. The per-class payment system leaves us financially vulnerable, often struggling to cover even basic expenses. Dealing with bureaucratic hurdles to claim reimbursements only adds to our financial stress, affecting our ability to focus on our professional duties*.”…. [AWKUMMMS-6].

Correspondingly, Daily wage teacher-7 examines:

“*I find myself trapped in a cycle of financial insecurity. The uncertainty of per-class payments makes it difficult to plan for the future or save for emergencies. Navigating through administrative red tape to access reimbursements adds an additional layer of frustration and anxiety*.”…. [AWKUMFMS-7].

Additionally, Daily wage teacher-8 investigates:

“*I feel the weight of economic instability on a daily basis. Despite our hard work, the per-class arrangement fails to provide a stable income, leaving us constantly worried about financial stability. The bureaucratic hurdles in claiming reimbursements only exacerbate our financial woes, leading to feelings of frustration and helplessness*.”…. [UOMMPHD-8].

Furthermore, daily wage teacher-9 views:

“*I struggle to make ends meet on a daily basis. The reliance on per-class payments often means that our income fluctuates, making it challenging to budget or save. Dealing with administrative delays in reimbursement claims adds to our financial strain, impacting our overall wellbeing*.”…. [UOSFMS-9].

Equally, daily wage teacher-10 debates:

“*I feel like I'm constantly juggling financial obligations. The uncertainty of per-class payments leaves us vulnerable to financial instability, unable to predict our earnings from month to month. The bureaucratic hurdles in claiming reimbursements only add to the stress, affecting our morale and dedication to our profession*.”…. [GUMMS-10].

Economic instability emerged as a dominant challenge for daily wage teachers, with the per-class payment system often failing to meet basic needs. Bureaucratic delays in reimbursements further exacerbate financial strain, undermining morale and professional dedication.

Focus group discussions revealed that financial stress leads to lifestyle compromises. One participant noted:

“*We have to think twice before spending on essentials, let alone saving for emergencies. The irregularity of payments makes budgeting a constant struggle*.”

Focus group discussions highlighted variations based on age and marital status, with younger teachers often facing greater instability due to a lack of additional income sources, whereas older, married teachers described the strain on family dynamics.

Sub-themes such as “Payment Irregularities,” “Bureaucratic Challenges,” and “Demographic Variations in Financial Impact” provide a nuanced understanding of these economic challenges. Addressing these issues requires systemic reforms, such as standardized payment structures and efficient administrative processes, to enhance financial security and morale. Focus group discussions (FGDs) reinforced the themes of financial instability, payment irregularities, and bureaucratic challenges revealed in the semi-structured interviews. However, FGDs also emphasized the collective frustration and solidarity among daily wage teachers, providing a broader understanding of how these issues affect the group dynamics.

Participants in FGDs consistently described irregular and insufficient payments as a major stressor. They shared collective experiences of struggling to budget for basic expenses and coping with the unpredictable nature of their income.

“*We all feel trapped in a cycle of financial uncertainty. Sometimes, months go by without payment, and we have no backup. It's like we are living from class to class*.” (FGD, AWKUM, September 2023)

This sentiment aligned with the individual accounts from interviews, where teachers highlighted their inability to meet basic needs or plan for the future. However, FGDs revealed a collective sense of shared vulnerability, which was less prominent in individual interviews.

Participants agreed that bureaucratic hurdles for reimbursements worsened their financial stress. Many expressed frustration over delays caused by inefficient administrative systems.

“*Navigating the system is exhausting. It's not just one of us—everyone here has faced the same hurdles. You feel like you're begging for what you're owed*.” (FGD, UOM, September 2023)

Unlike the interviews, which focused on individual frustrations, FGDs underscored a collective perception of neglect by university administration, suggesting systemic rather than isolated inefficiencies.

FGDs provided nuanced insights into how economic challenges varied by age and marital status. Younger participants expressed acute financial insecurity due to limited supplementary income sources, while older participants with families highlighted the strain on household budgets and relationships.

“*For us younger teachers, there's no backup—no savings, no family support. Older colleagues have families to support them, but the stress is still unbearable for everyone*.” (FGD, UOS, September 2023)

This demographic insight complemented interview findings but emphasized shared experiences over individual differences.

While interviews highlighted personal struggles with financial instability, FGDs brought out a collective sense of frustration and helplessness, creating a stronger emphasis on systemic failings.

“It's not just one person's problem. The whole system is broken. We all know someone who's thought about quitting because they can't survive on this.” (FGD, GU, September 2023)

Themes from FGDs largely aligned with semi-structured interviews, particularly regarding financial instability and bureaucratic delays. However, FGDs emphasized collective sentiments, adding depth to the analysis. The discussions revealed a shared sense of solidarity among participants, highlighting the systemic nature of their challenges and the urgent need for policy reforms to address these economic hardships.

### Academic Challenges

Daily wage teachers face academic challenges, with their qualifications overlooked, hindering career progression. Stagnation persists as their expertise is disregarded, making it difficult to secure permanent positions or pursue academic advancements. Reluctance to grant no-objection certificates adds to their professional entrapment.

In this context, Daily wage teacher-11 asserts:

“*I feel like my expertise is undervalued and overlooked. Despite my academic qualifications, I'm stuck in a cycle of professional stagnation. The lack of recognition and opportunities for advancement leave me feeling trapped in my career*.”…. [ICPUFPHD-11].

Likewise, Daily wage teacher-12 advocates:

“*I advocate for recognition of our academic qualifications. It's disheartening to see our expertise disregarded, hindering our professional growth. Without opportunities for advancement or acknowledgment of our skills, we feel trapped in our current positions*.”…. [ICPUMPHD-12].

In addition, Daily wage teacher-13 contends:

“*I contend with a sense of professional entrapment. Despite our qualifications, we're overlooked for permanent positions. The reluctance of universities to grant us no-objection certificates further limits our career opportunities, perpetuating a cycle of stagnation*.”…. [UOCMMS-13].

In the same fashion, daily wage teacher-14 proclaims:

“*I proclaim the need for fair treatment and recognition of our academic qualifications. Our experiences are valuable, yet universities fail to acknowledge them. Without opportunities for career advancement, we're left feeling trapped in our roles*.”…. [UOCFMS-14].

Similarly, daily wage teacher-15 defends:

“*I defend our right to academic recognition and advancement. It's unjust to overlook our qualifications and expertise. Without opportunities for career progression and acknowledgment of our skills, we're confined to a cycle of professional stagnation*.”…. [HUMFPHD-15].

Academic challenges faced by daily wage teachers stem from the undervaluation of their qualifications and a lack of career progression opportunities. Their expertise is often overlooked, limiting their ability to secure permanent positions or pursue academic advancements.

Focus group discussions highlighted systemic barriers. One participant remarked:

“*No matter how qualified we are, our contributions are dismissed. There's a constant struggle for acknowledgment*.”

Another noted the reluctance of universities to issue No-Objection Certificates (NOCs):

“*Without an NOC, pursuing better opportunities or further studies becomes almost impossible*.”

Focus group participants highlighted that younger teachers feel disillusioned about the profession, while senior teachers expressed frustration over years of unacknowledged service. Sub-themes such as “Recognition of Expertise,” “Barriers to Advancement,” and “Institutional Practices Affecting Career Growth” add depth to the analysis.

Addressing these challenges requires universities to implement fair policies, acknowledge academic contributions, and streamline the issuance of NOCs to support career development. Focus group discussions (FGDs) provided collective insights into the academic challenges faced by daily wage teachers, emphasizing the undervaluation of qualifications, lack of career progression opportunities, and institutional barriers like the reluctance to issue No-Objection Certificates (NOCs). These discussions both aligned with and expanded upon themes identified in semi-structured interviews, highlighting shared struggles and systemic issues.

FGD participants unanimously expressed frustration over the lack of recognition for their academic qualifications and contributions. Despite their expertise, they felt undervalued and excluded from opportunities for professional growth.

“*No matter how qualified we are, our contributions are dismissed. It feels like the system doesn't value what we bring to the table*.” (FGD, UOC, October 2023)

This collective sentiment reinforced the individual accounts from interviews, where teachers described feeling trapped due to their qualifications being overlooked. However, FGDs revealed a broader sense of shared dissatisfaction among participants.

Participants in FGDs highlighted systemic obstacles that prevent them from advancing in their academic careers. These include limited opportunities for promotion, exclusion from decision-making, and reluctance by institutions to support their professional development.

“We all feel like we're stuck in temporary roles with no clear path to advancement. It's not just one of us—it's a problem for all of us in these positions.” (FGD, ICPU, October 2023) “Without an NOC, pursuing better opportunities or further studies becomes almost impossible. It's a deliberate way to keep us confined.” (FGD, HUM, October 2023)

This aligns with interview findings, where teachers emphasized the professional stagnation caused by these barriers. However, FGDs brought out the collective frustration and a sense of institutional neglect.

The reluctance of universities to issue NOCs was a recurring theme in FGDs. Participants viewed this as a significant constraint that limited their mobility and career development. Senior participants expressed frustration over years of unacknowledged service, while younger teachers shared feelings of disillusionment.

“Younger teachers are already questioning whether this profession is worth it. And for those of us who've been here for years, it's disheartening to see no progress despite our hard work.” (FGD, UOS, October 2023)

This theme added depth to the analysis by showing how institutional practices disproportionately affect different demographics within the group.

While interviews emphasized individual frustrations with career stagnation, FGDs highlighted a collective sense of being trapped in a system that undervalues temporary staff. This collective perspective introduced a deeper understanding of how these challenges affect the group as a whole.

“*It's not just me or one of us—it's a structural issue. The system is designed to keep us in these roles without acknowledging our qualifications or contributions*.” (FGD, UOM, October 2023)

Themes from FGDs closely aligned with those from interviews, particularly regarding undervaluation of expertise and barriers to advancement. However, FGDs emphasized the shared struggles and systemic nature of these challenges, providing a more collective narrative. Addressing these academic challenges requires universities to recognize the qualifications of daily wage teachers, provide equitable opportunities for career progression, and streamline processes like issuing NOCs to support their professional growth.

### Administrative challenges

In the mentioned areas universities, daily wage teachers face administrative challenges marked by inefficiencies and arbitrary treatment. University authorities' exploitation and lack of transparency in administrative processes heighten vulnerability and erode trust in the institutional framework, leaving educators susceptible to mistreatment and manipulation.

In this connection, Daily wage teacher-16 probes:

“*I question the fairness of administrative practices. We're often subjected to arbitrary treatment and exploitation by university authorities. The lack of transparency and accountability undermines our trust in the institutional framework, leaving us vulnerable to mistreatment*.”…. [HUMMPHD-16].

Similarly, Daily wage teacher-17 demonstrates:

“*I experience firsthand the consequences of administrative inefficiencies. The arbitrary decisions and lack of transparency breed distrust among daily wage teachers. It's disheartening to see our concerns dismissed by university authorities*.”…. [KUSTFMS-17].

Correspondingly, Daily wage teacher-18 expands:

“*I witness the detrimental effects of administrative shortcomings. Daily wage teachers are treated unfairly, with little regard for our rights and wellbeing. The lack of transparency only exacerbates our vulnerability to manipulation and mistreatment*.”…. [KUSTMMS-18].

Also, daily wage teacher-19 delves:

“*I delve into the systemic issues plaguing administrative processes. The arbitrary treatment and lack of accountability create a culture of exploitation. Without transparency, daily wage teachers are left powerless, eroding our trust in the institutional framework*.”…. [UOMFMS-19].

Equally, daily wage teacher-20 inspects:

“*I inspect the disparities in administrative practices. The arbitrary decisions and lack of transparency leave us at the mercy of university authorities. This undermines our professional dignity and contributes to a sense of disillusionment within the academic community*.”…. [UOSMPHD-20].

Administrative inefficiencies and arbitrary treatment from university authorities exacerbate the challenges faced by daily wage teachers. These issues undermine trust in institutional frameworks, leaving educators vulnerable to exploitation.

Focus group discussions revealed that teachers often feel manipulated by administrative systems. One participant noted:

“*Decisions are made without consulting us, and we're rarely informed about policy changes that directly affect our roles*.”

Another added:

“*The lack of accountability creates a power imbalance, making us feel voiceless in addressing grievances*.”

Gendered nuances emerged in focus group discussions, with female teachers reporting additional challenges, such as discriminatory administrative practices and inadequate support systems. Sub-themes like “Transparency Deficits,” “Exploitation of Vulnerability,” and “Gender Disparities in Administrative Treatment” provide a richer understanding of these issues.

Institutional reforms emphasizing transparency, accountability, and inclusive decision-making are crucial to addressing these challenges and restoring trust. The focus group discussions revealed shared frustrations among daily wage teachers regarding administrative challenges in universities. These issues align with sentiments expressed in semi-structured interviews but highlight additional collective perspectives and nuances.

Participants collectively emphasized the lack of transparency and accountability in administrative practices, which mirrors concerns voiced in interviews. One participant noted:

“*We are never consulted on decisions that directly impact our roles. Policies are changed without warning, leaving us confused and unprepared*.” (FGD, UOM, November 2023). Another added: “*There's no system to address our concerns. It feels like everything happens behind closed doors, and we're just expected to comply*.” (FGD, KUST, November 2023).

A recurring theme in both interviews and FGDs was the feeling of exploitation. While interviews highlighted individual struggles, FGDs shed light on a collective sense of powerlessness:

“*We all feel like pawns in a larger system. There's no one to advocate for us, and the administration takes advantage of our vulnerable status*.” (FGD, UOS, November 2024). Another participant remarked:

“*Our temporary status is used as leverage. They know we can't afford to lose these jobs, so they exploit us without consequences*.” (FGD, HUM, November 2023).

FGDs uncovered gender-specific challenges, with female teachers reporting discriminatory practices and inadequate support systems. This nuanced perspective was less pronounced in interviews but emerged strongly in group discussions. One female participant shared:

“*For women, the challenges are double. We face the same lack of transparency and also deal with dismissive attitudes and discrimination*.” (FGD, UOM, November 2024). Another added:

“*There's no proper system for addressing issues unique to female staff. We're left to navigate these challenges alone*.” (FGD, KUST, November 2023).

The FGDs reinforced many of the findings from semi-structured interviews, particularly regarding administrative inefficiencies, arbitrary decision-making, and lack of transparency. However, FGDs brought out a collective dimension to these issues, as reflected in comments such as:

“*It's not just one of us; the entire group feels neglected and exploited by the system*.” (FGD, UOS, November 2023).

While individual interviews emphasized personal frustration and vulnerability, FGDs highlighted broader systemic issues and the collective impact on morale. A participant remarked:

“*Interviews focus on our individual experiences, but as a group, it's clear these problems are systemic. Everyone is affected, not just a few of us*.” (FGD, HUM, November 2023).

The FGDs underscore the urgent need for institutional reforms to address transparency deficits, exploitation, and gender disparities. By creating systems of accountability and inclusive decision-making, universities can foster trust and provide a supportive environment for daily wage teachers, ensuring their contributions are valued and respected.

### Psychological challenges

Daily wage teachers in Khyber Pakhtunkhwa, Pakistan, endure psychological challenges stemming from the cumulative impact of social, economic, academic, and administrative hurdles. Anxiety, depression, and uncertainty prevail as these educators contend with the daunting realities of their professional predicament, taking a toll on their psychological wellbeing.

Likewise, Daily wage teacher-21 explores:

“*I find myself overwhelmed by the cumulative challenges we face. The constant stress, uncertainty, and lack of stability in our profession take a significant toll on our mental wellbeing. It's a constant battle to maintain a sense of resilience*.”…. [GUFPHD-21].

Meanwhile, Daily wage teacher-22 examines:

“*I delve into the psychological impact of our precarious situation. The tension and anxiety are palpable as we navigate through social, economic, and administrative hurdles. Depression becomes a common companion in our struggle*.”…. [UOMMPHD-22].

Similarly, Daily wage teacher-23 explicates:

“*I articulate the profound sense of uncertainty that permeates our professional lives. The constant instability and lack of security breed anxiety and hopelessness among daily wage teachers. It's a constant battle to maintain our mental resilience*.”…. [UOPMPHD-23].

Correspondingly, daily wage teacher-24 notes:

“*I recognize the toll that our professional predicament takes on our psychological wellbeing. The pervasive sense of uncertainty and the constant struggle for recognition and stability contribute to feelings of anxiety and despair*.”…. [UOSFMS-24].

Finally, daily wage teacher-25 explains:

“*I experience firsthand the psychological challenges we face as daily wage teachers. The stress, anxiety, and uncertainty are constant companions as we navigate through the complexities of our professional lives. It's a struggle to maintain our mental health amidst the daunting realities we confront*.”…. [UOMMMS-25].

The cumulative impact of social, economic, academic, and administrative challenges takes a significant toll on the psychological wellbeing of daily wage teachers. Anxiety, depression, and uncertainty are pervasive, hindering resilience and overall mental health.

Focus group discussions highlighted the psychological strain resulting from professional instability. One participant noted:

“*The constant uncertainty about our jobs leads to sleepless nights. It's hard to maintain a positive outlook when you're always on edge*.”

Another shared:

“*The stress is overwhelming, especially when balancing professional struggles with personal responsibilities*.”

Demographic variations were apparent, with younger teachers describing heightened anxiety about career prospects, while older teachers expressed frustration over long-term instability. Sub-themes like “Emotional Impact of Job Insecurity,” “Coping Mechanisms,” and “Demographic Differences in Psychological Strain” enrich the analysis.

Addressing these psychological challenges requires institutions to provide mental health resources, foster job security, and create an environment that prioritizes the wellbeing of educators. Focus group discussions (FGDs) provided a collective lens into the psychological challenges faced by daily wage teachers in Khyber Pakhtunkhwa universities. The discussions highlighted the pervasive stress, anxiety, and uncertainty experienced due to job insecurity, economic instability, and lack of professional recognition. While semi-structured interviews captured individual experiences, FGDs emphasized shared struggles and the collective impact on mental wellbeing.

Participants consistently highlighted the mental strain caused by job instability. The unpredictability of contracts and lack of assurance about continued employment generated anxiety and sleeplessness.

“*The constant uncertainty about our jobs leads to sleepless nights. It's hard to maintain a positive outlook when you're always on edge*.” (FGD, GU, October 2023)

This theme aligned with individual interviews, where teachers described a pervasive sense of professional uncertainty. FGDs, however, emphasized the shared nature of this psychological strain, with participants collectively expressing the emotional toll of their precarious employment.

Despite the challenges, participants discussed various coping mechanisms, including relying on peer support and focusing on short-term achievements to maintain resilience.

“*We often lean on each other for emotional support. It's one of the few things that keep us going*.” (FGD, UOM, October 2023)

“*I try to focus on the small wins, like when a student acknowledges my efforts. It helps me get through the tougher days*.” (FGD, UOS, October 2023)

While interviews highlighted individual struggles, FGDs shed light on the communal strategies teachers employ to cope with psychological challenges, emphasizing the importance of social support networks.

FGDs revealed demographic variations in the psychological impact of job insecurity. Younger teachers expressed heightened anxiety about their career prospects, while older teachers voiced frustration over years of stagnation and unacknowledged service.

“*For us younger teachers, it's hard to imagine building a future in this profession. The lack of stability makes it nearly impossible to plan ahead*.” (FGD, UOM, October 2023)

“*We've been in these roles for so long, and it feels like nothing ever changes. The frustration builds over time, knowing our efforts are overlooked*.” (FGD, UOP, October 2023)

This nuanced perspective complements interview findings by highlighting how psychological challenges manifest differently across age groups.

While semi-structured interviews focused on individual experiences of stress and anxiety, FGDs underscored the collective nature of these challenges. The discussions also provided richer insights into coping mechanisms and demographic differences, which were less prominent in the interviews.

“*The stress is overwhelming, especially when balancing professional struggles with personal responsibilities*.” (FGD, UOS, October 2023)

“*We all feel the weight of uncertainty and instability. It's a constant battle to stay mentally strong*.” (FGD, GU, October 2023)

FGDs reinforced and expanded upon themes identified in interviews, emphasizing the shared psychological toll of precarious employment. Addressing these challenges requires systemic reforms, such as providing mental health resources, fostering job security, and recognizing the contributions of daily wage teachers. Institutional support is essential to mitigate the emotional impact and create a more supportive environment for these educators (see Table 2).

**Table 2:** Key challenges and themes in the professional lives of daily wage teachers: illustrative perspectives.

**Code**	**Theme**	**Illustrative quote**
Peer ostracization	Social Challenges	“I often feel like an outsider, barely acknowledged by my peers.”
Student perceptions	Social Challenges	“The stigma around being a 'temporary teacher' creates a barrier to forming meaningful relationships.”
Gendered exclusion	Social Challenges	“Female teachers face greater scrutiny and fewer networking opportunities.”
Payment irregularities	Economic Challenges	“The per-class payment system leaves us financially vulnerable.”
Bureaucratic challenges	Economic Challenges	“Delays in reimbursements only add to our financial stress.”
Demographic variations	Economic Challenges	“Younger teachers face greater instability due to a lack of additional income sources.”
Recognition of expertise	Academic Challenges	“Despite my academic qualifications, I'm stuck in a cycle of professional stagnation.”
Barriers to advancement	Academic Challenges	“The lack of recognition and opportunities for advancement leave me feeling trapped in my career.”
Institutional practices	Academic Challenges	“Universities are reluctant to issue No-Objection Certificates, limiting our career growth.”
Transparency deficits	Administrative Challenges	“We're often subjected to arbitrary treatment and exploitation by university authorities.”
Exploitation of vulnerability	Administrative Challenges	“The lack of transparency and accountability undermines trust in the institutional framework.”
Gender disparities	Administrative Challenges	“Female teachers report discriminatory administrative practices and inadequate support systems.”
Emotional impact	Psychological Challenges	“The constant instability and lack of security breed anxiety and hopelessness among daily wage teachers.”
Coping mechanisms	Psychological Challenges	“It's a constant battle to maintain a sense of resilience.”
Job insecurity	Psychological Challenges	“Uncertainty about our jobs leads to sleepless nights.”

## Discussion

### Theme 1: social challenges

Daily wage teachers face significant social challenges, often perceived as outsiders within academic institutions. This sense of exclusion aligns with the phenomenon of “peripheralization,” where contingent faculty feel marginalized and undervalued (Curtis et al., 2016; Gates, 2015). Such marginalization hinders meaningful collaboration and professional development, diminishing job satisfaction and institutional commitment (Barak and Levin, 2002; Wynants and Dennis, 2018). Addressing these issues requires institutional initiatives to foster inclusion and recognition, which are critical for enhancing engagement and retention.

### Theme 2: economic challenges

Financial instability remains a predominant challenge for daily wage teachers, reflecting broader trends in contingent faculty experiences. Per-class payment models and delayed reimbursements exacerbate financial insecurity, echoing findings by Hillier (2020) and Maynard and Joseph (2008). This unpredictability undermines financial planning and overall quality of life, highlighting the need for equitable compensation structures (Rhoades et al., 2024). Furthermore, administrative inefficiencies in reimbursement processes add to their economic burden, resonating with Andro's (2021) emphasis on the detrimental effects of systemic delays on job satisfaction.

### Theme 3: academic challenges

Daily wage teachers report a lack of recognition for their qualifications and limited opportunities for advancement, reflecting systemic barriers identified by Kezar and Maxey (2016). This undervaluation negatively impacts professional identity and career growth, perpetuating a cycle of 'professional entrapment' where institutional policies restrict career progression (Birdsell Bauer, 2011; Kilfoye, 2015). Reforming these structures to offer equitable pathways for advancement is crucial to empowering contingent faculty and fostering their contributions to academia.

### Theme 4: administrative challenges

Administrative inefficiencies, such as a lack of transparency in institutional processes, emerged as critical issues. These inefficiencies undermine trust in leadership and adversely affect morale, aligning with findings by Lyke (2019) and Kakabadse and Bank (2018). Participants in this study emphasized the arbitrariness of institutional policies, which mirrors Mech's (2017) observations on the exploitation of contingent faculty. Institutional reforms promoting fairness and accountability are necessary to improve contingent faculty's professional experiences and trust in governance.

### Theme 5: psychological challenges

The precarious nature of temporary employment profoundly impacts the psychological wellbeing of daily wage teachers, contributing to stress, anxiety, and feelings of hopelessness (Crowder, 2016; Peake et al., 2018). The heightened psychological distress reported in this study aligns with findings by Kezar et al. (2019) and Campbell (2022), which emphasize how job insecurity amplifies mental health challenges. Institutions must prioritize proactive measures, such as mental health support and enhanced job stability, to mitigate these adverse effects.

This study integrates key findings with existing literature, emphasizing the multifaceted challenges faced by daily wage teachers. It highlights the interplay between administrative inefficiencies, economic instability, and psychological distress, offering a nuanced understanding of their experiences. Unique to this study is the exploration of how administrative hurdles exacerbate both financial and mental health challenges, adding depth to the discourse. Future research should focus on evaluating institutional strategies to promote equity, inclusion, and wellbeing for contingent faculty.

## Conclusion

**Figure 2:**
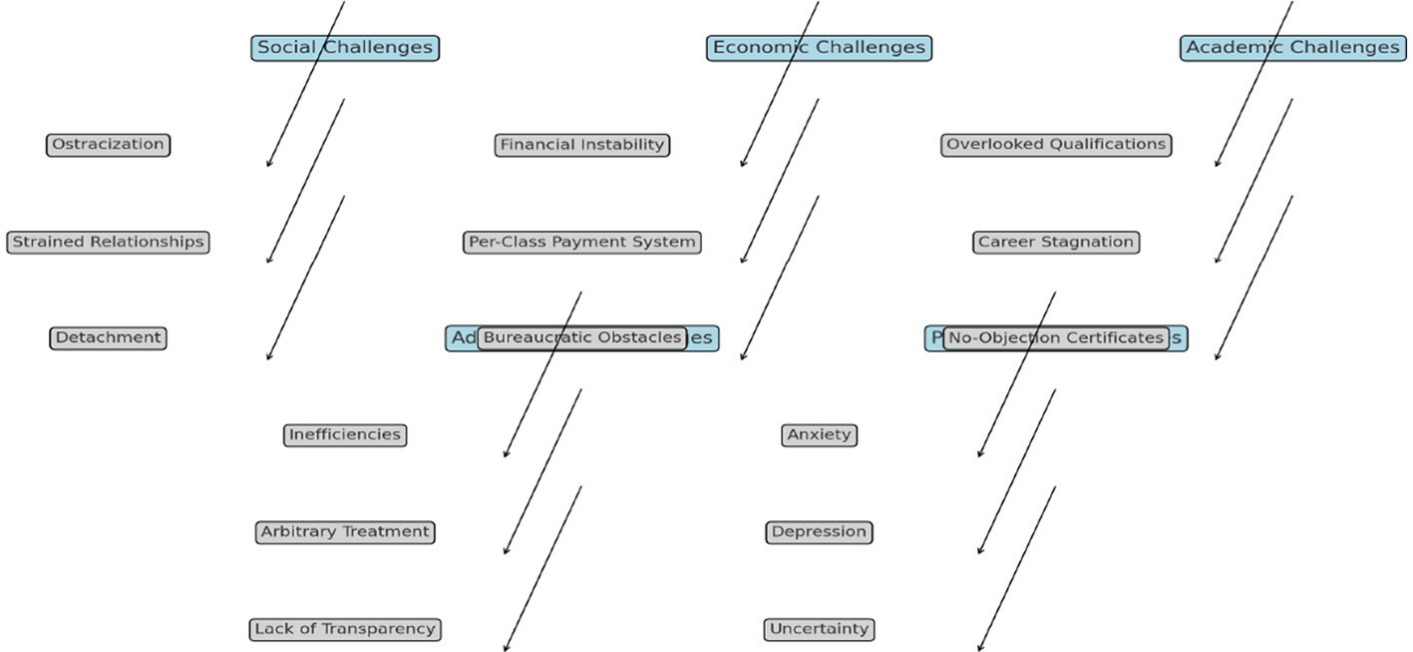
Summary of the results.

This study highlights the complex challenges faced by daily wage teachers in Khyber Pakhtunkhwa, Pakistan, revealing how social exclusion, financial instability, limited career progression, and administrative inefficiencies collectively undermine their wellbeing and professional growth. By integrating these experiences with existing research, the study underscores the pressing need for systemic reforms to address these structural barriers (see Figure 2).

A key contribution of this research is its detailed examination of how intersecting challenges affect daily wage teachers' psychological resilience and institutional engagement, offering a nuanced understanding of their lived experiences. The findings advocate for policy reforms that prioritize equitable compensation, transparent administrative processes, and mental health support to foster a more inclusive and supportive academic environment.

Addressing these issues is not only a moral imperative but also a strategic necessity for enhancing the quality and sustainability of higher education. Ensuring the dignity, wellbeing, and professional advancement of all educators is crucial to building a resilient academic community that can effectively contribute to societal progress.

## Policy implications

This study highlights actionable measures to address the systemic challenges faced by daily wage teachers in Khyber Pakhtunkhwa, Pakistan. The recommendations focus on fostering inclusivity, ensuring economic stability, and improving institutional practices to enhance their professional and personal wellbeing.

### Formalize employment practices

Universities should implement formal contracts to ensure job security, timely compensation, and access to benefits such as healthcare and pensions, directly addressing financial instability and workplace uncertainty.

### Foster inclusivity

Institutions must adopt measures to integrate daily wage teachers into academic communities, recognizing their contributions and reducing feelings of marginalization.

### Career pathways

Clear, equitable opportunities for career advancement should be established, acknowledging the qualifications and expertise of daily wage teachers to mitigate professional stagnation.

### Streamline administrative processes

Simplifying and enhancing transparency in administrative procedures can reduce bureaucratic delays, fostering trust and reducing stress among educators.

### Mental health support

Establishing mental health resources and counseling services tailored to daily wage teachers can help address the psychological toll of precarious employment conditions.

### Advocacy and representation

Mechanisms for teacher advocacy and inclusion in decision-making processes can ensure their voices are heard and their concerns addressed.

### Continuous monitoring and evaluation

Regular evaluation of institutional policies and practices will ensure that interventions remain effective, evidence-based, and aligned with the needs of daily wage teachers.

## Limitations and gap for future research

One of the key limitations of this study is its focus on the immediate challenges faced by daily wage teachers, without exploring the long-term effects of the policies and interventions implemented to address these issues. While the study provides valuable insights into the current struggles of these educators, it does not assess how the implementation of support mechanisms, such as formal employment contracts, professional development programs, and psychological support services, influences their long-term wellbeing and career progression. This limitation opens the door for future research to investigate the sustained impact of these interventions over time, offering a deeper understanding of their effectiveness in enhancing the professional lives of daily wage teachers.

Moreover, the study does not explore the effectiveness of specific support mechanisms in detail. Future research could focus on evaluating the success of social integration initiatives and mental health services tailored to daily wage teachers. By investigating the real-world impact of these programs, future studies can provide evidence-based recommendations for improving institutional support structures for daily wage educators.

Another limitation is the lack of exploration into the intersectionality of challenges faced by different demographic groups among daily wage teachers. The study did not investigate how factors such as age, gender, educational background, and years of experience may intersect to shape the unique challenges faced by various subgroups within this population. Future research could examine these intersections, exploring how the needs of different demographic groups vary and identifying targeted approaches to address the distinct challenges they face.

Finally, the scalability of successful interventions is not explored in this study. Given the diverse contexts within academic institutions, the effectiveness of interventions may vary across different universities or regions. Future research should evaluate the scalability and adaptability of successful support mechanisms, allowing for the development of targeted, sustainable strategies to address the complex and evolving needs of daily wage teachers across various institutional settings.

## Data Availability

The original contributions presented in the study are included in the article/supplementary material, further inquiries can be directed to the corresponding author.

## Appendix

**Table A1:** Detailof participants.

**Coding with explanations**	**Age**	**Gender**	**Education**	**Experience**
UOPMMS-1 (University of Peshawar Male MPhil Degree)	33	M	MS	2 Years
UOPMPHD-2 (University of Peshawar Male PhD Degree)	37	M	PhD	5 Years
UOPFMS-3 (University of Peshawar Female MPhil Degree)	30	F	MS	4 Years
UOAMMS-4 (University of Agriculture Male MPhil Degree)	33	M	MS	4 Years
UOAFPHD-5 (University of Agriculture Female PhD Degree)	44	F	MS	9 Years
AWKUMMMS-6 (Abdul Wali Khan University Mardan Male MPhil Degree)	36	M	MS	3 Years
AWKUMFMS-7 (Abdul Wali Khan University Mardan Female Mphil Degree)	34	F	MS	5 Years
UOMMPHD-8 (University of Malakand Male PhD Degree)	45	M	PhD	10 Plus Years
UOSFMS-9 (University of Swat Female Mphil Degree)	34	F	MS	5 Years
GUMMS-10 (Gomal University Male Mphil Degree)	36	M	MS	4 Years
ICPUFPHD-11 (Islamia College Peshwar University Female PhD Degree)	40	F	PhD	9 Years
ICPUMPHD-12 (Islamia College Peshwar University Male PhD Degree)	44	M	PhD	10 Years
UOCMMS-13 (University of Chitral Male Mphil Degree)	33	M	MS	4 Years
UOCFMS-14 (University of Chitral Female Mphil Degree)	36	F	MS	5 Years
HUMFPHD-15 (Hazara University Mansehra Female PhD Degree)	44	M	PhD	10 Plus Years
HUMMPHD-16 (Hazra University Mansehra Male PhD Degree)	47	M	PhD	10 Years
KUSTFMS-17 (Kohat University of Science and Technology Female Mphil Degree)	34	F	MS	6 Years
KUSTMMS-18 (Kohat University of Science and Technology Male Mphil Degree)	36	M	MS	7 Years
UOMFMS-19 (University of Malakand Female Mphil Degree)	35	F	MS	6 Years
UOSMPHD-20 (University of Swat Male PhD Degree)	43	M	PhD	9 Years
GUFPHD-21 (Gomal University Female PhD Degree)	34	F	PhD	7 Years
UOMMPHD-22 (University of Malakand Male PhD Degree)	45	M	PhD	10 Years
UOPMPHD-23 (University of Peshawar Male PhD Degree)	47	M	PhD	10 Plus Years
UOSFMS-24 (University of Swat Female Mphil Degree)	34	F	MS	6 Years
UOMMMS-25 (University of Malakand Male Mphil Degree)	36	M	MS	7 Years
